# Direct evidence of plant consumption in Neolithic Eastern Sudan from dental calculus analysis

**DOI:** 10.1038/s41598-024-53300-z

**Published:** 2024-02-21

**Authors:** Giusy Capasso, Dulce Neves, Alessandra Sperduti, Emanuela Cristiani, Andrea Manzo

**Affiliations:** 1 Department of Cultural Heritage, University of Padua, Padua, Italy; 2 Research Centre for Anthropology and Health, University of Coimbra, Coimbra, Portugal; 3 Department of History, Anthropology, Religions, and Performing Arts, Sapienza University of Rome, Rome, Italy; 4 Bioarchaeology Service, Museum of Civilizations, Rome, Italy; 5 Department of Asian, African and Mediterranean Studies, University ‘L’Orientale’, Naples, Italy; 6 DANTE - Diet and ANcient TEchnology Laboratory, Department of Oral and Maxillo-Facial Sciences, Sapienza University of Rome, Rome, Italy

**Keywords:** Plant domestication, Climate-change ecology

## Abstract

The Neolithic communities of Eastern Sudan combined intensive pastoralism with plant exploitation as their main subsistence strategies. However, to date, it remains unclear which plant species were part of the human diet during the Neolithic. This contribution presents direct data on plant consumption in Eastern Sudan from the Early to Late Neolithic, obtained through the analysis of microdebris inclusions in the dental calculus of 37 individuals, integrated by dentoalveolar pathology analysis of 78 individuals, from the sites UA53 (4th millennium BCE) and Mahal Teglinos (3rd–2nd millennium BCE), located in the Gash Delta/Kassala region. Dental calculus inclusions indicate a diverse intake of cereals, legumes, and tubers during the Middle Neolithic, thus supporting the hypothesis of high reliance on plant resources. Dentoalveolar pathologies, possibly related to the consumption of carbohydrate-rich foods, have also been recorded. For the Late Neolithic, consistent with the shift towards aridity that occurred in the Middle/Late Holocene, dental calculus exclusively indicates the exploitation of sorghum and tubers—species well adapted to arid conditions—showing how the Neolithic communities modified their subsistence in response to environmental changes. Evidence of plant processing techniques, such as cooking/heating, was also revealed from the dental calculus analysis.

## Introduction

Until recently, the prevailing hypothesis, based on the well-preserved archaeozoological record, was that Sudanese Neolithic economies were mostly based on pastoralism, whereas the role of plants was seldom recognized and poorly supported by direct evidence^1–5^. More recently, Salvatori & Usai^5^ emphasised the importance of exploring the relationships between different regional forms of pastoralism and other types of food production to enhance our comprehension of the processes and patterns of biocultural adaptation to the environment.

Studies on Northern and Central Sudan have provided solid evidence of the exploitation of domesticated and wild plant species, including gathered fruits^4,6,7^. In some cases, it has been suggested that the plants may also have played a symbolic role in the funerary rituals^4,8^.

The importance of plant exploitation in Eastern Sudan is well recognized, thanks to archaeobotanical and genetic studies, that have highlighted the central role of the Gash Delta/Kassala region as the place where sorghum was first domesticated in the 4th millennium BCE and as a gateway for the spread of African crops during the 2nd millennium BCE^9,10^. Indeed, due to the abundance of water and soil resources in the areas between the Gash and the Atbara rivers, which are particularly suitable for agricultural exploitation, in sub-Saharan Africa, Eastern Sudan has been singled out for its role in the history of African crops.

In addition to the rich archaeobotanical evidence from the Gash Delta/Kassala region^11–16^, the presence of grindstones/tools in archaeological assemblages from the region also suggests the adoption of economic strategies based on plant exploitation^17–20^. To further support this hypothesis, dental analyses carried out on some Mesolithic and Neolithic series pointed to a worsening in oral health during the Neolithic, as a possible consequence of increased consumption of carbohydrates-rich foods^21,22^. Indeed, a decline in oral health is one of the most commonly reported negative effects of the Neolithic transition worldwide^23^ and several studies have shown a positive correlation between dentoalveolar disease and the consumption of highly cariogenic wild and domesticated plant foods.

Our knowledge of plant food exploitation in Eastern Sudan is primarily based on archaeobotanical and archaeological evidence, which provides insights into past vegetation, ecology, and agricultural practices, supporting indirect hypotheses about potential food sources. Therefore, a direct assessment of which plant species were included in human diets, and a more consistent exploration of plants and crop processing techniques still need to be carried out systematically. To fill this gap, this study investigates plant processing and consumption in Eastern Sudan by analysing the archaeological dental calculus from individuals from two different graveyards, dated from the Early (4th millennium BCE) to the Late Neolithic (2nd millennium BCE). In the last decade, the human mineralized plaque has proven its potential to provide broader, long-term perspectives on dietary patterns of past populations, offering a more immediate snapshot of individual dietary patterns and specific food item consumption, hence offering a unique glimpse into short-term subsistence choices^24,32^.

As has been widely demonstrated in previous studies, during the formation of dental calculus, biomolecules, and inclusions—such as starches, pollen, phytoliths, spores, diatoms, or fibres—can be trapped and preserved within its mineral matrix, protected from chemical destruction and other diagenetic changes^24–26^. Therefore, dental calculus allows us to access direct evidence of ancient diets, oral microbiomes, local environments, cultural behaviours, and extra-masticatory daily life tasks using teeth as a ‘third hand’^6,27–34^.

Overall, this paper aims (a) to contribute to an understanding of the plants selected for food during the Neolithic in Eastern Sudan, also concerning data already known for other areas of Sudan; (b) to provide evidence for plant and crop processing activities; (c) to assess whether changes in dietary composition during the Neolithic could potentially be linked to the increase in oral pathologies observed across the Neolithic phases. While previous studies of archaeological dental calculus from prehistoric Northern and Central Sudan have provided important data on past subsistence in these areas^4,6,7,35–37^, this contribution adds new information on the role of plants in economic strategies in Neolithic Eastern Sudan, being the first study undertaken for this area.

### Archaeobotanical evidence from Eastern Sudan

The available archaeobotanical data from Eastern Sudan suggest that the exploitation of domestic plants in this area began in the 4th millennium BCE^9^. Archaeobotanical evidence and genetic studies^15,38,39^ have shown that, after a long period of collecting and harvesting of wild morphotypes (*Sorghum verticilliflorum*), the Eastern Sudanese savannah including southern Atbai and the Gash Delta/Kassala region, became a centre for the origins of sorghum pre-domestication from this period onwards. Indeed, the site Khashm el Girba 23 (KG23), provided the earliest evidence of the domesticated sorghum (*S. bicolor*) dated to 3700–2900 BCE^15,16^. Further evidence for domesticated sorghum dating from the 3rd millennium BCE comes from the sites of Jebel Moya, south of Khartoum, and Mahal Teglinos (K1), in the Gash Delta^13,40^. The domestication of sorghum in Eastern Sudan probably resulted from a period of human‐mediated selection that fixed the loss of the natural dispersal mechanism of seeds^14–16^.

However, if sorghum cultivation was well‐established in the Southern Atbai by 3000 BCE, the exploitation of wild forms continued in later periods^16^, as indicated by the presence of morphologically wild sorghum (*S. verticilliflorum*) dating to the 2nd millennium BCE, recorded as pottery impressions at the site Mahal Teglinos (K1)^11,14,15^. According to Haaland^41^, this was probably due to continuous cross-pollination processes between domesticated and wild forms. These processes resulted in intermediate forms of wild sorghum growing in the vicinity of cultivated populations, which may also have been exploited as part of a mixed subsistence strategy combining both cultivation and collection of wild specimens using baskets or collecting from the ground^16,42^. Indeed, modern ethnographic studies point out the importance of wild grass consumption in the central Sahel, where a complex of typical savannah grasses known as *kreb* is collected and used to prepare foods such as bread, flour, couscous, and beer^43–45^.

Due to its strategic geographical location, Eastern Sudan also played a crucial role in the diffusion of *Sorghum bicolor* and pearl millet (*Pennisetum glaucum*) outside the country, by connecting the African Sahel with Egypt, the Red Sea regions, and the Arabian Peninsula^14,46^. Indeed, from the 2nd millennium BCE, the region became part of an extensive long-distance trade network as it served as the northern overland gateway to Punt, the land mentioned in the Egyptian texts from the 5th Dynasty (ca. 2500–2350 BCE), from which the Egyptians imported various African resources, including aromatic gums, animals, animal skins, ebony, ivory, and precious metals^9,10,47,48^. In addition to sorghum and pearl millet, the macrobotanical remains and plant impressions recorded on ceramics dating from the 3rd to the 1st millennium BCE in the area of Gash Delta/Kassala region also provide evidence for the exploitation of other plant species such as *Adansonia digitata*, *Ziziphus spina-christi*, *Celtis integrifolia*, *Vigna unguiculata*, *Grewia bicolor*, *Triticum durum/aestivum*, *Hordeum vulgare*, *Rottboellia cochinchinensis*, and several millets (*Setaria, Eleusine, Paspalum, Echinochloa*)^12,13,49^.

### The archaeological sites

**Figure 1:**
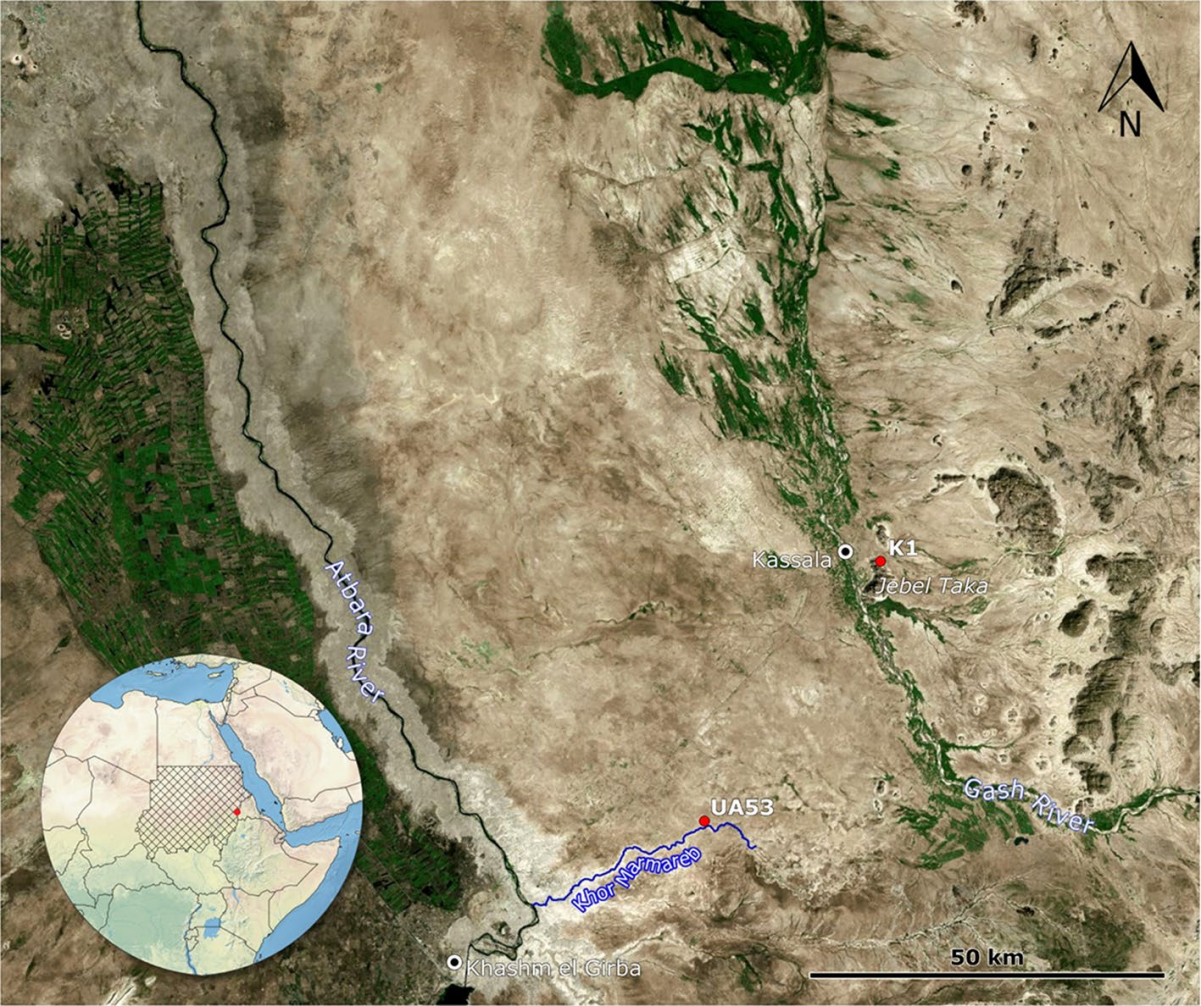
Image showing the location of the sites Upper Atbara 53 (UA53) and Mahal Teglinos (K1), in the Gash Delta/Kassala region, Eastern Sudan. Image courtesy of Stefano Costanzo, created with QGIS 3.4 (QGIS Development Team, 2019) using public domain satellite imagery datasets retrieved through the QGIS plugin QuickMapServices (NextGIS, 2019).

Since 2010, the IAEES (Italian Archaeological Expedition to Eastern Sudan) has been carrying out excavations in the area between the Gash and Atbara rivers. Among the sites investigated, Upper Atbara 53 (UA53) and Mahal Teglinos (K1), both located in the Gash Delta/Kassala region, allowed us to cover a chronological sequence spanning from the Early to the Late Neolithic, corresponding to the Butana Group, the Gash Group, and the Jebel Mokram Group in the regional sequence^9^ (Fig. 1). These sites yielded 152 burials—single or double—with the individuals placed in different positions and with different body orientations. The osteological sample comprises 163 individuals. All age groups, including infants, had access to a formal burial throughout the whole period considered^21,22^.

#### Site Upper Atbara 53 (UA53)

UA53 is a Butana Group site located along the Khor Marmareb^9^. The site yielded several shell middens dating to the early 4th millennium BCE, probably resulting from the intensive exploitation of land snails. At the site, 16 single tombs dating to the Late Butana Period (last centuries of the 4th millennium BCE) were excavated^50^. The individuals were placed in a contracted position with different orientations and with grave goods consisting only of personal ornaments, mainly lip plugs, sometimes made of Red Sea shells^9^. The anthropological analyses allowed the identification of 15 adults and an 8–9-year old child^22^.

Archaeological excavations at other sites related to the Butana Group suggest that the economy of this culture was based on wild game such as antelopes, swine, and elephants, as well as on fishes, freshwater shellfish, and land snails^9,51^. Archaeozoological data support the importance of cattle exploitation in the later phases^9^. In addition, domesticated and morphologically wild sorghum were exploited^49^.

#### Site Mahal Teglinos (K1)

Located near the modern city of Kassala, Mahal Teglinos (K1) is one of the largest Gash Group sites. Twelve radiocarbon dates from this site indicate a chronology ranging from the mid-3rd to the first part of the 2nd millennium BCE for Gash Group culture^9^. The archaeological excavations in sector K1 XII of the Western graveyard yielded 106 burials, of which 98 were single and 8 double burials, with individuals lying in a supine position. The anthropological sample comprises 114 individuals, with 88 adults (77.2%) and 26 (22.8%) subadults^22^. The presence of exotic goods at the site and in some tombs proves that Eastern Sudan was involved in inter-regional exchange networks with Upper Nubia, Egypt, the Red Sea regions or coasts, and Yemen during the late 3rd–2nd millennium BCE^9,10^.

During this phase, both wild and domesticated animals were exploited, including freshwater snails, fishes, reptiles, birds, mammalian game, and livestock^9,51^. The archaeobotanical record from K1 includes remains of both wild and domesticated plants, such as sorghum, barley, wheat, baobab seeds, etc.^14,49,50^.

More recently, in the southern sector of the western graveyard, trench K1 XIV yielded 30 burials, dating to the late Gash Group and Jebel Mokram phase (early 2nd—early 1st millennium BCE). In contrast to the earlier Gash Group phase, the individuals (20 adults and 10 subadults) were placed exclusively in a tightly contracted position^22^.

Some aspects of the material culture, the characteristics of the settlements, and their distribution across the region suggest profound transformations in the lifestyle and economy associated with the transition from the Gash to the Jebel Mokram period. During the latter phase, as a possible consequence of the arid transition that occurred from the Middle/Late Holocene^52,53^, in the grazing areas between the Gash and the Atbara rivers, the number of sites increased dramatically while their extent decreased. Moreover, the thinness of the stratigraphic deposits suggests their identification as short-term seasonal camps^9,10^ The archaeozoological and archaeobotanical evidence from the Jebel Mokram sites suggests that in addition to the high relevance on pastoralism^9,51,54^, plant resources such as pearl millet (*Pennisetum glaucum*), ziziphus (*Ziziphus spina-christi*), cowpea (*Vigna unguiculata*), and sorghum (*Sorghum bicolor*) were also exploited^9,11,49^.

## Results

Of the 37 individuals analysed, 22 yielded microdebris. Specifically, dental calculus yielded starch grains from 16 individuals (43.2%), phytoliths from 3 individuals (8.1%), other plant remains (i.e. fibres and plant tissues) from 17 individuals (45.9%), wood fragments from 5 individuals (13.5%), and charcoal from 3 individuals (8.1%) (see Table 1).

**Table 1:** Microdebris from archaeological dental calculus samples from Early Neolithic (site Upper Atbara 53, UA53), Middle Neolithic (site Mahal Teglinos, K1 XII), and Late Neolithic (site Mahal Teglinos, K1 XIV) in Eastern Sudan. Damaged/modified starches; Starch morphotype 1 (Triticeae); Starch morphotype 2 (Fabaceae); Starch morphotype 3 (tubers); Starch morphotype 4 (Poaceae); Phytoliths; Other plant remains (fibres and vegetal tissues); Spore/pollen grains; Charcoal; Wood; the symbol “ > ” is used when starch granules cannot easily be counted.

ID Lab	Chronology	Damaged/modified starches	Starch morphotype I (Triticeae)	Starch morphotype II (Fabaceae)	Starch morphotype III (tubers)	Starch morphotype IV (Poaceae)	Phytoliths	Other plant remains	Spore/pollen grains	Charcoal	Wood
SD_01	Early Neolithic	1				1		1			
SD_02	Early Neolithic					1		3			
SD_05	Late Neolithic				1	2		4			1
SD_06	Late Neolithic					1				1	
SD_07	Late Neolithic					3		2			
SD_09	Middle Neolithic		1	2				2	1		
SD_10	Middle Neolithic	1	6		1			1			
SD_11	Middle Neolithic						1	1	2		
SD_14	Middle Neolithic		1								
SD_17	Middle Neolithic	1					2	7			1
SD_18	Middle Neolithic		> 50								
SD_23	Middle Neolithic							10			
SD_24	Middle Neolithic	2	6			3		4	1		
SD_25	Middle Neolithic		> 50					1			
SD_26	Middle Neolithic							4			
SD_27	Middle Neolithic							2		1	1
SD_30	Middle Neolithic		1	3				2			
SD_33	Middle Neolithic							2			
SD_34	Middle Neolithic			20		1					1
SD_35	Middle Neolithic							1			
SD_36	Middle Neolithic		> 50			1		4			1
SD_37	Middle Neolithic	1						2		1	
	Total	6	> 165	25	2	12	3	53	4	3	5

### Starch granules

**Figure 2:**
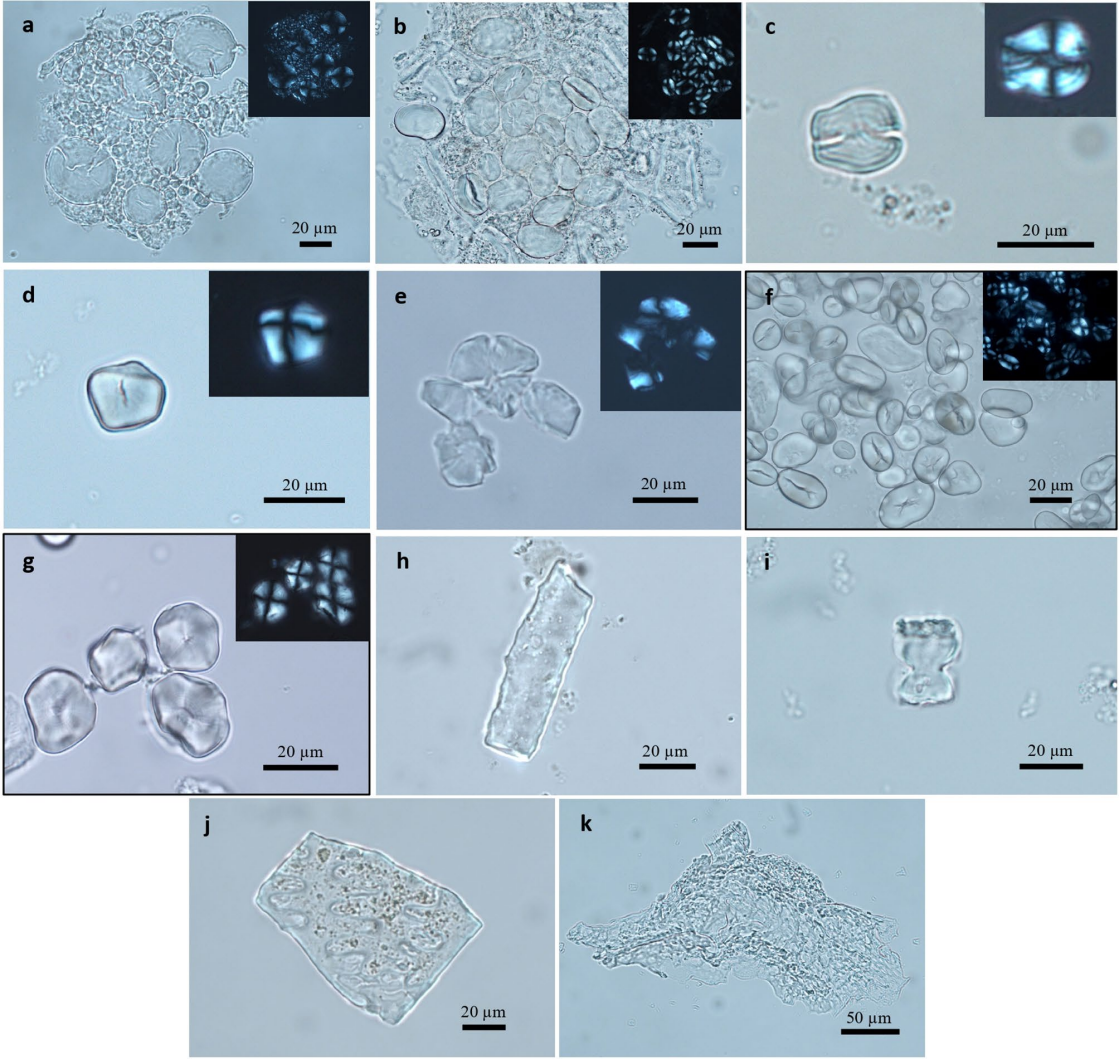
Starch granules, phytoliths, and vegetal tissue fragments from archaeological dental calculus of individuals from sites Upper Atbara 53 (UA53) and Mahal Teglinos (K1) in Eastern Sudan. (**a**) Morphotype 1: Triticeae starch granules from SD_25 (site K1 XII, Middle Neolithic). (**b**) Morphotype 2: Fabaceae starch granules from SD_34 (site K1 XII, Middle Neolithic). Note that the starches are still embedded in the calculus matrix. (**c**) Starch granule associated with tubers, showing similarities with the *Encephalartos* genus, from SD_10 (site K1 XII, Middle Neolithic). (**d**) Morphotype 4: Poaceae starch granule from SD_05 (site K1 XIV, Late Neolithic). (**e**) Modified starch granule from SD_01 (site UA53, Early Neolithic). (**f**) Starch granules of modern *Vigna unguiculata* (Fabaceae). (**g**) Starch granules of modern *Sorghum bicolor* (Poaceae). (**h**) Epidermal long-cell phytolith from SD_11 (site K1 XII, Middle Neolithic). (**i**) Bilobate short-cell phytolith commonly associated with the Poaceae from SD_17 (site K1 XII, Middle Neolithic). (**j**) Interdigitate phytolith from SD_24 (site K1 XII, Middle Neolithic). (**k**) Fragment of spongy parenchyma from SD_23 (site K1 XII, Middle Neolithic).

Calculus samples from Eastern Sudan yielded both undamaged and damaged starch grains. The samples with identifiable starch granules relate to 14 individuals from all the chronological phases considered. The grains were classified into four different morphotypes as follows:

*Morphotype 1*. Starch grains (N_tot_ =  > 165) belonging to this morphotype were observed in the dental calculus of eight individuals from the Middle Neolithic sample (see Table 1). The grains are often lodged in the amyloplast. This morphotype is characterised by a bimodal distribution, involving the presence of large round to oval granules dimensionally variable (19.9–63.1 μm), occasionally showing lamellae (A-Type) and small spherical granules (< 10 μm) without visible lamellae (B-Type). Both types show a central hilum and a defined centric extinction cross under cross-polarised light (see Fig. 2) These features and the bimodal distribution are consistent with Triticeae starches (Poaceae family)^29–33,55,56^. A-Type grains display diagnostic features while smaller B-Type grans are rarely diagnostic to taxa^57^. In our archaeological population, we observed an unimodal distribution characterised by a reduced quantity of A-Type grains and a large quantity of B-Type grains. Some of the A-type grains show morphological alterations probably related to plant processing, cooking/heating, or enzymatic digestion (salivary amylase)^58–63^.

*Morphotype 2.* Starch grains (N_tot_ = 25) belonging to this morphotype were identified in the dental calculus of three individuals from the Middle Neolithic sample (see Table 1). The granules are still associated with endosperm tissues, as a possible consequence of the partial processing for cooking purposes. The large grains (12.5–33 μm) have an ovoid shape, centric hilum, rugose surface, visible lamellae, and a defined extinction cross with four or more well-defined arms (see Fig. 2). The morphological characteristics are diagnostic of Fabaceae starch grains. In particular, the characteristics observed fall within the Faboideae subfamily of Fabaceae^7,29,64^ which includes East African legume species such as the hyacinth bean (*Lablab purpureus*) and cowpea (*Vigna unguiculata*) included in the archaeobotanical record from Eastern Sudan^7,64^.

*Morphotype 3*. Starch grains (N_tot_ = 2) belonging to this morphotype were identified in two individuals from the Middle Neolithic (n = 1) and the Late Neolithic sample (n = 1) (see Table 1). The granules are characterised by a polyhedral shape with two deep notches on the surface, a central hilum, and visible lamellae. A well-defined extinction cross with four straight arms is visible under cross-polarised light (see Fig. 2). This morphotype reaches a maximum width of 19.8 μm. These features, consistent with tuber starch granules, show similarities with the *Encephalartos* genus (Zamiaceae family)^64,65^.

*Morphotype 4*. Starch grains (N_tot_ = 12) belonging to this morphotype were identified in eight individuals from the Early Neolithic (n = 2), Middle Neolithic (n = 3), and Late Neolithic samples (n = 3) (see Table 1). This morphotype is characterised by a polyhedral/sub-polyhedral shape (10–20.8 μm) and includes both single grains or two aggregated grains. The grains have a wrinkled surface, flat facets, centric hilum, and radiating fissures (Y-fissures). Under polarized light, the extinction cross with four straight arms is well visible, while lamellae are not visible (see Fig. 2). These morphological characteristics fall within the Panicoidae subfamily of Poaceae, which include the so-called “big millets” such as *Sorghum bicolor* or *Pennisetum glaucum*^7,29,66,67^. Both these millets are included in the archaeobotanical record from Eastern Sudan^13–16^.

#### Modified/damaged starches

Unidentifiable modified/damaged grains (N_tot_ = 6) were recorded in the Early (n = 1) and Middle Neolithic (n = 5) samples (see Table 1; see Fig. 2). These modifications are probably due to mechanical processing (e.g., grinding, pounding), cooking/heating, or fermentation^58–61,68^.

### Phytoliths

Phytoliths were scarce in the calculus samples. They were found only in three individuals from the Middle Neolithic series (see Table 1; see Fig. 2). Among these, we recorded a bilobate short-cell phytolith (Site K1 XII: Tomb 28), which is commonly associated with Panicoideae grasses (family Poaceae)^66,69^.

### Generic micro remains

Various other plant structures were also recorded. Micro-remains of probable non-dietary origin include fungal spores/hyphae, charcoal, fibres, and wood fragments. Some undifferentiated plant tissues (e.g., spongy parenchyma) were also identified^70^ (see Fig. 2).

### Dentoalveolar pathologies

Dental caries was observable in all 78 individuals, for a total of 1173 teeth examined, while the scoring of *ante mortem* tooth loss (AMTL) and abscesses was possible in 62 individuals.

**Figure 3:**
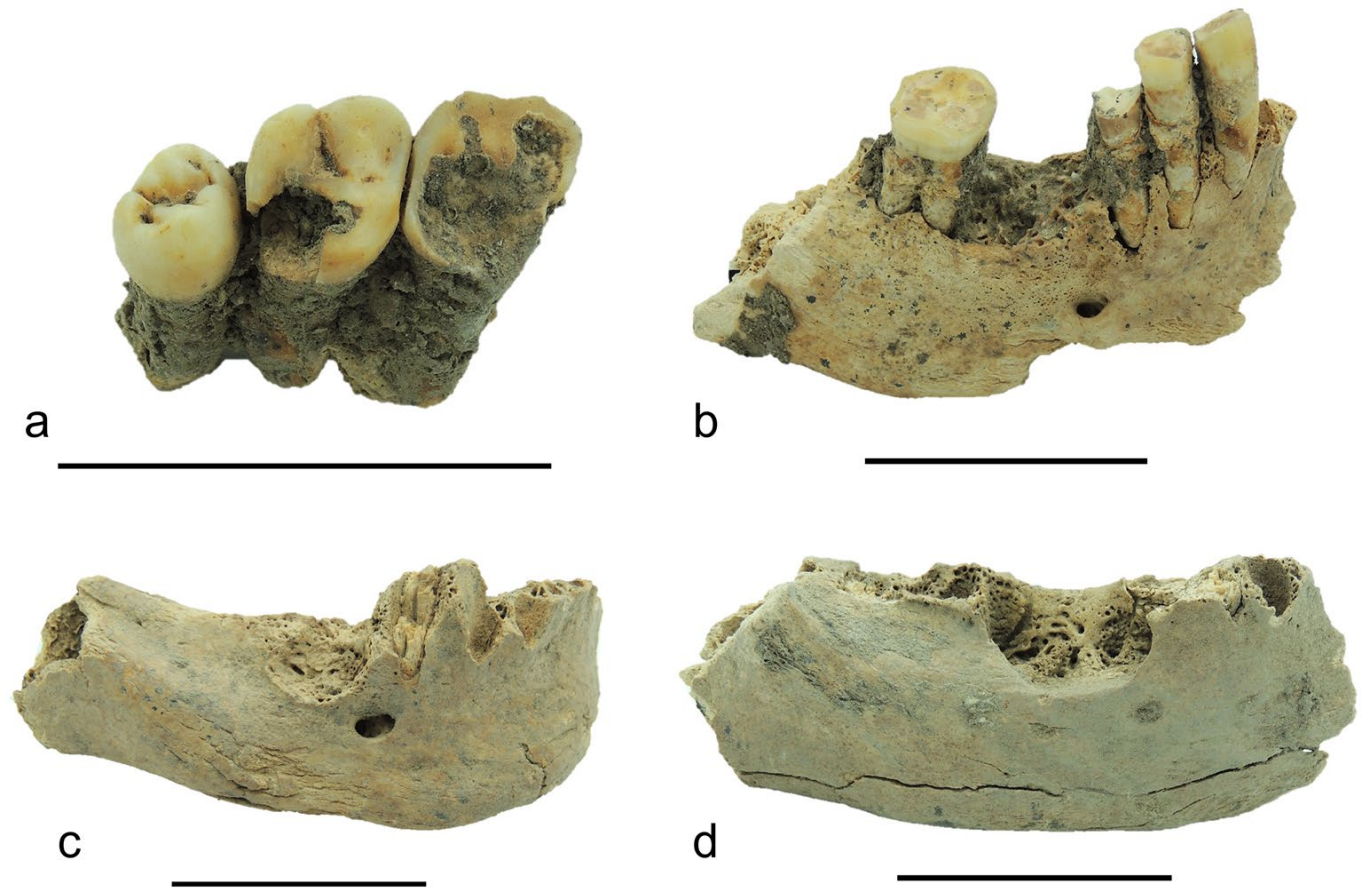
Dentoalveolar pathologies recorded from the site Mahal Teglinos (K1) in Eastern Sudan. (**a**) Dental caries affecting the upper right second molar (URM2) in a 40 + -year-old female individual (Tomb 52, Middle Neolithic). (**b**) Ante mortem tooth loss (AMTL) of the lower right first molar in a 40 + -year-old male individual (Tomb 23, Middle Neolithic). (**c**) Abscess and AMTL of the lower right first molar in a 40 + -year-old male individual (Tomb 30, Middle Neolithic). (**d**) Abscess and AMTL of the lower right first and second molars in a 40 + -year-old female individual (Tomb 111, Middle Neolithic).

Dentoalveolar pathologies were recorded in 41% of the individuals considered (32/78), with the Middle Neolithic group being more affected than the others (Early Neolithic = 1/13: 7.7% affected; Middle Neolithic = 28/57: 49.1% affected; Late Neolithic = 2/8: 25% affected) (see Supplementary Table S3 online; see Fig. 3). Dental caries preferentially affected the posterior teeth, with no differences in the degree and localisation of lesions between the different groups. AMTL was recorded in 8 individuals only, all from the Middle Neolithic series. Regarding oral pathology affecting the bone, a high percentage of the individuals show alveolar resorption (66/78, 84.6%), while periapical abscesses were found in only three individuals from the Middle Neolithic sample (see Supplementary Table S3 online). As expected, in all samples, oral pathologies preferentially affected individuals over 30 years of age. Furthermore, in the Middle Neolithic sample, females were more affected than males (F: 19/31, 61.3% vs M: 9/26, 34.6%) (see Supplementary Table S3 online).

## Discussion

Dental calculus analysis revealed a diverse intake of grains in the diet, including cereals, legumes, and tubers in Neolithic Eastern Sudan. These results are consistent with the archaeobotanical evidence from the region and allow us to support the hypothesis of increased reliance on agriculture from the Neolithic onwards. Similar trends have been reported for Northern and Central Sudan, where archaeological dental calculus and isotopic analyses also suggest the exploitation of the same resources^6,7,36,37^.

Calculus inclusions indicate that cereals were widely exploited in Eastern Sudan during the Neolithic (see Table 1), in line with what has also been documented in Northern and Central Sudan^7–35^. Indeed, the majority of samples containing starch grains (14/16) yielded grains associated with Triticeae and Panicoidae. In addition, one of the three phytoliths recorded from the Middle Neolithic sample (Site K1 XII: Tomb 28) is associated with the grasses of the Paniceae.

In particular, dental calculus yielded grains associated with *Sorghum bicolor* and *Pennisetum glaucum*. These plants, well-adapted to arid conditions—the latter being the most drought-tolerant among the cereals—are widespread in Africa, representing one of the major food sources in arid and semi-arid areas^66^. The archaeobotanical record from the Gash Delta/Kassala region suggests that the consumption of sorghum, already documented for the Early and Middle Neolithic periods, continued—and most likely even increased—during the Late phase, included within the agropastoral adaptive system of the Jebel Mokram group, perhaps also thanks to its suitability for arid conditions^9^. Consistent with this evidence, the dental calculus yielded *Sorghum* and *Pennisetum* grains for all the phases considered (see Table 1).

Furthermore, legume consumption is documented during the Middle Neolithic. To date, legumes have been rarely documented in Sudanese archaeobotanical assemblages due to poor preservation conditions caused by wet/arid cycles and high microbial activity^5^. However, consistent with our results, macro remains from the Kassala region include *Vigna unguiculata*, commonly known as cowpea^12,49^. This legume, with high nutritional value, was first introduced from West African countries to the western Sudanese regions of Kordofan and Darfur^71^. The inclusion of legumes into the diet of Neolithic Sudanese populations has already been demonstrated in Northern and Central Sudan^4,7^.

The analysis indicates that the Neolithic diet also included tubers, a nutritious source of carbohydrates. The inclusion of tubers in the diet of Sudanese Neolithic groups has already been suggested^7^. Among the cycads (Zamiaceae family), the genus *Encephalartos* is entirely endemic to Africa. In fact, phylogenetic studies have shown that this genus, which originated in southern Africa, was then dispersed within the continent through vicariance and dispersal events^65^. In particular, *Encephalartos mackenziei* is endemic to Sudan^72^. In addition, *Cyperus rotundus* has been documented in Central Sudan, at the multi-period site of Al Khiday, where it was supposed to have been used also for non-nutritional purposes, given its aromatic or medicinal qualities^6^. Since these tubers show a remarkable ability to adapt to arid conditions^65,73,74^ their presence in the Late Neolithic assemblage, together with sorghum, fits well with the climatic transition to aridity recorded in the region starting from the Middle/Late Holocene onwards^52,53^.

In several cases, starch grains displaying morphological changes were recorded, suggesting the possibility of plant food processing, e.g. by cooking/heating or grinding, which resulted in the loss of starch’s characteristic morphological features, in some cases precluding their identification. Charcoal remains in dental calculus may further support the hypothesis of food cooking practices. Processing by cooking is highly required to make legumes more digestible and nutritious^75,76^. Smoke inhalation, dry (roasting), and wet (heating in water) cooking have already been documented in Central Sudan, where damaged starches and charcoal fragments have been retrieved by dental calculus as well^6^. Moreover, it is important to consider that mastication, involving the action of amylase—a digestive enzyme present in human saliva—may also play a role in the morphological alteration of the starch grains^62,63^.

In addition to cooking techniques, the evidence of starch granules which are exceptionally well preserved in groups and still included in the amyloplast could also indirectly indicate the low level of processing for the preparation of starchy foods. Furthermore, the presence of phytoliths in dental calculus may be caused by several extra-dietary sources, such as accidental inhalation or dust in the environment generated by the use of grasses in a variety of activities and uses, such as flooring and kindling^58,59,61^. This evidence could also be linked to the grinding stones found in the archaeological assemblages from the Kassala region, which further suggest the role of plants in daily life activities^17–20^. Outside Eastern Sudan, grinding stones recovered from other Mesolithic and Neolithic Sudanese sites have also been suggested to have been used for processing plant foods^41^. This hypothesis also seems to be supported by the phytolith analysis of quern stones from the Wadi Howar area, in Western Sudan, which attest to the exploitation of chloridoid and panicoid grasses (4300 cal. BCE)^77^. The cemetery of Ghaba provided evidence for some form of processing of wild grasses, including the use of very sharp tools in systematic and parallel movements^4^. Furthermore, the presence of plant fibres in the dental calculus of individuals from the Middle Neolithic site (K1) could be related to the non-masticatory use of teeth, as also suggested by the dental wear patterns displayed by some of the individuals^21,22^. This aspect will be investigated in further analysis.

The results of dental calculus analysis were complemented by dental paleopathology, which provided additional information on the dietary trends of prehistoric populations in Eastern Sudan. The dental analysis indicated high rates of dentoalveolar pathology in Eastern Sudan during the Neolithic. This trend is consistent with the almost worldwide reported decline in oral health during the Neolithic^23^. Indeed, numerous studies have shown a positive correlation between the prevalence of periodontal disease and the consumption of highly cariogenic wild and domesticated plant foods due to the progressive demineralisation of dental tissues caused by the acids produced during the fermentation of food particles^78–84^. This study suggests that in addition to the consumption of agricultural carbohydrates, the increase in oral pathologies recorded in Eastern Sudan during the Neolithic may also be related to the consumption of wild grasses, which are rich in fermentable carbohydrates. Fruits may also have played a role in the onset of oral pathologies, as they are rich in sugar and quite cariogenic, especially when eaten dried. The macrobotanical assemblage from Eastern Sudan includes charred and desiccated fruit stones of the nettle tree (*Celtis integrifolia*), ziziphus (*Ziziphus spina-christi*), baobab tree (*Adansonia digitata*), white raisin (*Grewia bicolor*), and date palm (*Phoenix dactylifera*)^49,85,86^. The consumption of edible wild fruits has also been documented in Neolithic sites in Central Sudan^36^.

Dental analysis showed that in the Middle Neolithic females were more affected by oral pathology than males (61.3% vs 36%). Similar differences have been recorded in many prehistoric populations^87–89^ and bioanthropologists have generally proposed differences in diet and sexual division of labour to explain this phenomenon^90,91^. However, according to the clinical literature, a higher prevalence of caries in women could also be due to hormonal fluctuations at different stages of a woman's life, such as puberty, pregnancy, and menopause, that alter the biochemical composition of saliva and its flow rate, weakening its buffering and antimicrobial effects, thus negatively affecting women’ oral health^92–94^. No significant differences were recorded in dental calculus inclusions between the sexes during the Middle Neolithic phase, as we recorded starches and phytoliths in both males (n = 5) and females (n = 7).

This study falls within a line of research on dental calculus from prehistoric Sudan initiated in 2014 by the study conducted by Buckley and colleagues^6^ for the multi-period site of Al-Khiday, in Central Sudan. As mentioned above, several studies have been conducted over the years, covering Northern and Central Sudan, while this is the first study for Eastern Sudan.

The data obtained are of significant value due to the lack of δ^13^C and δ^15^N analysis, precluded by the poor collagen preservation in the recovered skeletal and dental remains from Eastern Sudan. In addition, even though if the application of analytical methods such as sequential thermal desorption–gas chromatography–mass spectrometry (TD–GC–MS) and pyrolysis–gas chromatography–mass spectrometry (Py–GC–MS) would have aided in starch identification, allowing the recognition the organic components of starches^6^, the large number of studies on dental calculus from different prehistoric Sudanese sites served as a reliable reference sample for our study. In addition, the extensive reference collection at the DANTE-Laboratory greatly facilitated the identification process.

## Conclusions

Through the examination of dental calculus, this study contributes to our understanding of dietary habits and subsistence strategies in prehistoric Eastern Sudan, providing new direct data on plant consumption from the Early to the Late Neolithic and allowing us to support the hypothesis of high agricultural dependence from the Neolithic onwards. In the absence of δ^13^C and δ^15^N analysis, dental calculus represented a unique opportunity to provide direct information on plant-based subsistence patterns in this region.

Firstly, the study highlights that a high diversity of plant resources was exploited, with a mixed diet including both C3 and C4 plants, such as cereals, legumes, and tubers, consistent with the archaeobotanical evidence from the Gash Delta/Kassala region and with what has been recorded for other areas of Sudan. In addition, the inclusion of legumes and tubers in the dental calculus indicates these resources played a role in the Neolithic diet despite a void in the archaeobotanical evidence for these plant resources from the region. Secondly, the presence of damaged starches and charcoal fragments in dental calculus indicates plant processing activities, such as heating/cooking and grinding. Thirdly, the study supports the hypothesis that high rates of oral pathology recorded in Eastern Sudan during the Neolithic may be related to the consumption of wild grasses and agricultural fermentable carbohydrates.

From a diachronic perspective, dental calculus exclusively indicates sorghum consumption for the earliest phase, while the greatest variability in plant resources is evident during the Middle Neolithic period. However, the exclusive presence of sorghum in the Early Neolithic sample could be due to the limited size of the sample available for this phase. For the Late Neolithic period, dental calculus exclusively revealed evidence for the consumption of sorghum and tubers. As these plant groups have been shown to adapt well to arid conditions, this evidence fits well with the climatic transition to aridity that occurred in the region from the Middle/Late Holocene onwards. Considering that during the same period, there was also a shift toward pastoralism, followed by an increase in the number of short-term seasonal camps, the exclusive exploitation of plant species well adapted to arid conditions can be seen as an additional indicator of the significant changes in the lifestyles and subsistence strategies of the Neolithic communities in response to landscape evolution during the Middle/Late Holocene. In particular, incorporating plant consumption into pastoralist diets represents a form of flexibility and adaptability that can help them withstand environmental and socio-economic change. The evidence for plant consumption provided by this study, combined with archaeological, archaeobotanical, and ecological data, contributes to a more comprehensive understanding of the strategies employed by Neolithic Sudanese pastoral societies to thrive in dynamic landscapes.

Finally, this study confirmed the great potential of dental calculus analysis to provide insights into different aspects of ancient societies and their biocultural adaptations.

## Methods

All the permits for this study were obtained from the National Corporation for Antiquities and Museums (NCAM), Khartoum, Sudan, as part of a research agreement established with the IAEES (Italian Archaeological Expedition to Eastern Sudan).

**Figure 4:**
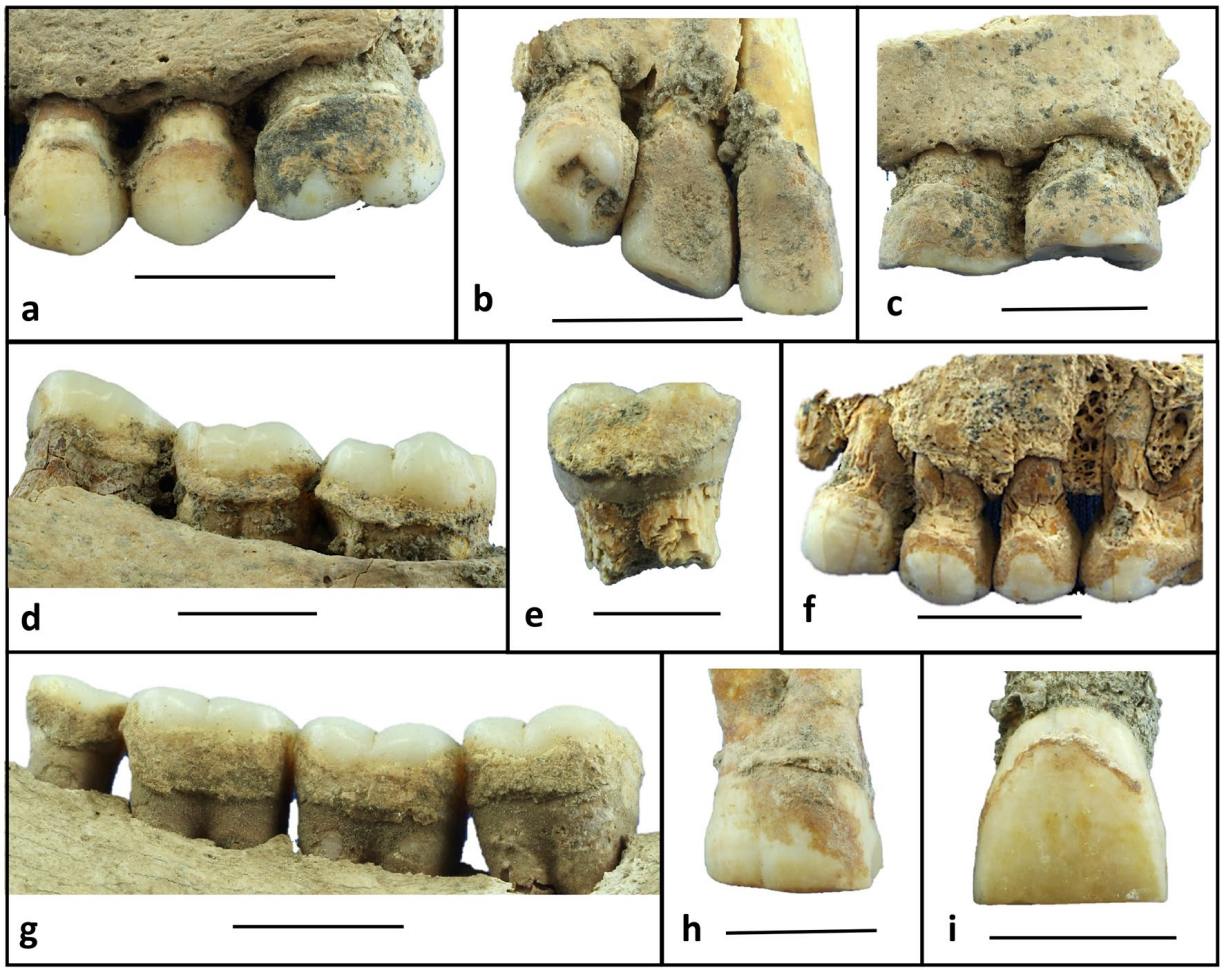
Archaeological dental calculus preserved on teeth from Neolithic sites Upper Atbara 53 (UA53) and Mahal Teglinos (K1) in Eastern Sudan. (**a**) Site K1 XII, Tomb 61. (**b**) Site K1 XII, Tomb 78 (Sk.1). (**c**) Site K1 XII, Tomb 73 (Sk.1). (**d**) Site K1 XII, Tomb 28. (**e**) Site K1 XII, Tomb 36. (**f**) Site K1 XII, Tomb 26. (**g**) Site K1 XIV, Tomb 8 (Sk.1). (**h**) Site K1 XII, Tomb 50. (**i**) Site K1 XII, Tomb 72. (Scale bar: 1 cm.).

Archaeological dental calculus was collected from 37 adult individuals from the UA53 site (4th millennium BCE, n = 4) and the full (3rd millennium BCE, n = 30) and late (2nd millennium BCE, n = 3) strata of the Mahal Teglinos (K1) site, comprising a total of 41 teeth of all tooth classes and both arches (see Supplementary Tables S1 and S2 online; see Fig. 4). Sex and age at death of the individuals were assessed following methods defined in the anthropological literature^95^.

To avoid damage to the teeth surfaces, the sampling, decontamination, and extraction procedures were carried out according to standard protocols as described by Fiorin and colleagues^96^ and Sabin and Fellow-Yates^97^ in an environmentally controlled room at the DANTE-Diet and Ancient Technology Laboratory of Sapienza University of Rome. The weight of the calculus removed from each tooth ranged from 1.4 to 2.8 mg. After removal, the samples were sealed in sterile Eppendorf tubes. A weak solution of 0.06 N of HCl was used to extract and mount the microfossils included in the calculus matrix. A solution of 50:50 glycerol and ultrapure water was used to allow for the rotation of the microfossils. Examination of the microfossils was carried out using a Zeiss IMAGER M2 compound polarised microscope (100–630 ×). The operators wore powder-free gloves during all phases.

Micro remains were described according to their morphological characteristics. Starches granules were grouped into morphotypes, based on their morphology, size, shape, presence of lamellae, hilum morphology, formation characteristics (i.e., simple or compound), cross features, cracks, and extinction cross characteristics under polarised light microscopy. Taxonomic identification was based on published literature^57,98,99^ and supported by the reference collection housed at the DANTE Laboratory, which comprises over 300 species of wild and domestic plants native to the Mediterranean region and Africa.

Phytoliths were described according to the International Code for Phytolith Nomenclature (ICPN) 2.0^100^ and identified using published literature^7,66,67,101^.

Dentoalveolar pathologies were recorded according to the methods by Buikstra and Ubelaker^102^. Only adult individuals with at least three observable permanent posterior teeth were considered for the analysis (N = 78 individuals. Early Neolithic site UA53: n = 13; Middle Neolithic site K1 XII: n = 57; Late Neolithic site K1 XIV: n = 8).

The microparticles extracted and analysed in this study are currently stored at the DANTE-Diet and Ancient Technology Laboratory of the Sapienza University of Rome, while the dental samples are stored at the University of Naples ‘L’Oriental’, Italy, available for future studies.

### Supplementary Information

Supplementary Information.

## Data Availability

All data generated or analysed during this study are included in this published article (and its Supplementary Information files).
