# Supplementary material for: Direct evidence of plant consumption in Neolithic Eastern Sudan from dental calculus analysis
Source: Sci Rep. 2024 Feb 21;14:4278. doi: 10.1038/s41598-024-53300-z (PMC10882051; doi:10.1038/s41598-024-53300-z)
Supplement: Supplementary file 1 — Supplementary Information. [file 41598_2024_53300_MOESM1_ESM.pdf]

Title: Direct evidence of plant consumption in Neolithic Eastern Sudan from dental calculus analysis

Authors: Giusy Capasso[1*], Dulce Neves[2-3], Alessandra Sperduti[4-5], Emanuela Cristiani[6**], Andrea Manzo[5]

1 Department of Cultural Heritage, University of Padua, Padua, Italy

2 Research Centre for Anthropology and Health, University of Coimbra, Coimbra, Portugal

3 Department of History, Anthropology, Religions, and Performing Arts, Sapienza University of Rome, Rome, Italy

4 Bioarchaeology Service, Museum of Civilizations, Rome, Italy

5 Department of Asian, African, and Mediterranean Studies, University of Naples "L'Orientale", Italy

6 DANTE - Diet and ANcient TEchnology Laboratory, Department of Oral and Maxillo-Facial Sciences, Sapienza University of Rome, Rome, Italy

*Corresponding author email: giusy.capasso@phd.unipd.it

**Co-corresponding author email: emanuela.cristiani@uniroma1.it

Supplementary Table S1: The dental sample by tooth types selected for dental calculus analysis.

Tooth type	n
**UI1** (Permanent Upper Central Incisor)	2
**UI2** (Permanent Upper Lateral Incisor)	1
**UP3** (Permanent Upper Third Premolar)	2
**UP4** (Permanent Upper Fourth Premolar)	1
**UM1** (Permanent Upper First Molar)	11
**UM2** (Permanent Upper Second Molar)	4
**LI1** (Permanent Lower Central Incisor)	1
**LI2** (Permanent Lower Lateral Incisor)	1
**LP3** (Permanent Lower Third Premolar)	4
**LM1** (Permanent Lower First Molar)	9
**LM2** (Permanent Lower Second Molar)	6

Supplementary Table S2: Samples considered for dental calculus analysis from sites Upper Atbara 53 (UA53) and Mahal Teglinos(K1), with the indication of site, grave, sex and age, chronology, and tooth sample.

Lab ID	Site	Grave	Sex	Age class	Chronology	Tooth sample
SD_01	UA 53 XII	1	F	20-30	Early Neolithic	LM1
SD_02	UA 53 XVII	2	F	20-30	Early Neolithic	LI2
SD_03	UA 53 XIX	1	M	30-40	Early Neolithic	LP3
SD_04	UA 53 XXI	1	M	40+	Early Neolithic	UP3
SD_05	K1 XIV	8 (SK.1)	F	30-35	Late Neolithic	LM1, LM2
SD_06	K1 XIV	10	F	20-30	Late Neolithic	UM1
SD_07	K1 XIV	3	M	20-25	Late Neolithic	UM2
SD_08	K1 XII-XIII	2	M	40+	Middle Neolithic	LP3
SD_09	K1 XII-XIII	3	F	40+	Middle Neolithic	UM1
SD_10	K1 XII-XIII	5	F	30-35	Middle Neolithic	UM1
SD_11	K1 XII-XIII	6	M	30-35	Middle Neolithic	LM2
SD_12	K1 XII-XIII	8	F	20-24	Middle Neolithic	LM1
SD_13	K1 XII-XIII	14	M	30-40	Middle Neolithic	LP3
SD_14	K1 XII-XIII	21	M	20-30	Middle Neolithic	UM2
SD_15	K1 XII-XIII	25	F	30-35	Middle Neolithic	UM1
SD_16	K1 XII-XIII	26	M	30-35	Middle Neolithic	UM1
SD_17	K1 XII-XIII	28	F	40+	Middle Neolithic	LM1, LM2
SD_18	K1 XII-XIII	30	M	40+	Middle Neolithic	UI1
SD_19	K1 XII-XIII	36	M	20-30	Middle Neolithic	LM1
SD_20	K1 XII-XIII	50	M	30-40	Middle Neolithic	UM2
SD_21	K1 XII-XIII	53	F	30-40	Middle Neolithic	UM1
SD_22	K1 XII-XIII	61	F	15-20	Middle Neolithic	UM1
SD_23	K1 XII-XIII	71	F	40+	Middle Neolithic	UP4
SD_24	K1 XII-XIII	72	M	30-35	Middle Neolithic	UI1
SD_25	K1 XII-XIII	73 (SK.1)	F	30-35	Middle Neolithic	UM1, UM2
SD_26	K1 XII-XIII	73 (SK.2)	M	40+	Middle Neolithic	LP3

SD_27	K1 XII-XIII	77	F	20-30	Middle Neolithic	LM1
SD_28	K1 XII-XIII	78 (SK.1)	F	20-24	Middle Neolithic	UI2
SD_29	K1 XII-XIII	78 (SK.2)	M	40+	Middle Neolithic	LM2
SD_30	K1 XII-XIII	84	F	40+	Middle Neolithic	UM1
SD_31	K1 XII-XIII	87	M	30-40	Middle Neolithic	UM1
SD_32	K1 XII-XIII	98	F	40+	Middle Neolithic	LM1
SD_33	K1 XII-XIII	100 (SK.1)	F	30-35	Middle Neolithic	LM2
SD_34	K1 XII-XIII	100 (SK.2)	F	20-30	Middle Neolithic	UM1
SD_35	K1 XII-XIII	101	F	40+	Middle Neolithic	LM1
SD_36	K1 XII-XIII	108	M	40+	Middle Neolithic	UP3
SD_37	K1 XII-XIII	116	F	30-40	Middle Neolithic	LM1, LM2

Supplementary Table S3: Results of the dental analysis on 78 individuals from Early Neolithic (n=13), Middle Neolithic (n=57), and Late Neolithic (n=8) Eastern Sudan. AMTL= *ante mortem* tooth loss.

Site	Grave	Sex	Age class	Caries	Abscess	AMTL	Affected	Alveolar resorption
UA 53 XII	T. 1	F	20-30	no	no	no	no	no
UA 53 XIII	1	ND	30-40	no	no	no	no	yes
UA 53 XV	1	F	20-30	no	no	no	no	no
UA 53 XV	2	ND	30-40	no	no	no	no	yes
UA 53 XV	3	ND	30-40	no	no	no	no	yes
UA 53 XVI	1	M	40+	no	no	no	no	yes
UA 53 XVII	1	F	20-30	no	no	no	no	no
UA 53 XVII	2	M	40+	yes	no	no	yes	yes
UA 53 XIX	1	ND	30-40	no	no	no	no	yes
UA 53 XIX	2	ND	40+	no	no	no	no	yes
UA 53 XIX	3	ND	30-40	no	no	no	no	yes
UA 53 XX	1	ND	20-30	no	no	no	no	no
UA 53 XXI	1	M	40+	yes	no	no	yes	yes
K1 XII	2	M	40+	yes	no	yes	yes	yes
K1 XII	3	M	40+	yes	no	no	yes	yes
K1 XII	5	M	30-40	no	no	no	no	yes
K1 XII	6	M	30-40	no	no	no	no	yes
K1 XII	8	F	20-30	no	no	no	no	no
K1 XII	9	F	40+	yes	yes	no	yes	yes
K1 XII	14	M	30-40	no	no	no	no	yes
K1 XII	15 (Sk.1)	F	30-40	yes	no	no	yes	yes
K1 XII	16	F	30-40	yes	no	no	yes	yes
K1 XII	17	F	30-40	yes	no	no	yes	yes
K1 XII	21	M	20-30	no	no	no	no	no
K1 XII	22	F	30-40	yes	no	no	yes	yes
K1 XII	23	M	40+	yes	no	yes	yes	yes
K1 XII	25	F	30-40	yes	no	no	yes	yes
K1 XII	26	M	30-40	yes	no	no	yes	yes
K1 XII	28	F	40+	yes	no	no	yes	yes
K1 XII	30	M	40+	yes	yes	yes	yes	yes

K1 XII	34	M	40+	no	no	yes	yes	yes
K1 XII	35 (Sk.1)	M	30-40	yes	no	yes	yes	yes
K1 XII	35 (Sk.2)	M	30-40	no	no	no	no	yes
K1 XII	37	M	30-40	no	no	no	no	yes
K1 XII	50	M	30-40	no	no	no	no	yes
K1 XII	52	F	40+	yes	no	no	yes	yes
K1 XII	53	F	30-40	yes	no	no	yes	yes
K1 XII	58	M	40+	yes	no	no	yes	yes
K1 XII	59	M	30-40	no	no	no	no	yes
K1 XII	60	F	40+	yes	no	no	yes	yes
K1 XII	61	F	20-30	no	no	no	no	no
K1 XII	63	M	40+	no	no	yes	yes	yes
K1 XII	65	F	40+	no	no	yes	yes	yes
K1 XII	66	M	30-40	no	no	no	no	yes
K1 XII	67	F	40+	no	no	no	no	yes
K1 XII	71	F	40+	no	no	no	no	yes
K1 XII	72	M	30-40	no	no	no	no	yes
K1 XII	73 (Sk.1)	F	40+	no	no	no	no	yes
K1 XII	73 (Sk.2)	M	30-40	no	no	no	no	yes
K1 XII	76	F	40+	no	no	no	no	yes
K1 XII	77	F	20-30	no	no	no	no	no
K1 XII	78 (Sk.1)	F	20-30	no	no	no	no	no
K1 XII	78 (Sk.2)	M	40+	no	no	no	no	yes
K1 XII	84	F	40+	no	no	no	no	yes
K1 XII	87	M	30-40	no	no	no	no	yes
K1 XII	88	M	30-40	no	no	no	no	yes
K1 XII	93	F	40+	yes	no	no	yes	yes
K1 XII	98	F	40+	yes	no	yes	yes	yes
K1 XII	100 (Sk.1)	F	30-40	yes	no	no	yes	yes
K1 XII	100 (Sk.2)	F	20-30	no	no	no	no	no
K1 XII	101	F	40+	yes	no	no	yes	yes
K1 XII	103	M	30-40	no	no	no	no	yes
K1 XII	106	F	40+	yes	no	no	yes	yes
K1 XII	107	F	40+	yes	no	yes	yes	yes
K1 XII	108	M	40+	no	no	no	no	yes
K1 XII	111	F	40+	no	yes	no	yes	yes
K1 XII	113 (Sk.1)	F	40+	no	no	no	no	yes
K1 XII	116	F	30-40	yes	no	no	yes	yes
K1 XII	117	M	30-40	no	no	no	no	yes
K1 XII	119	F	40+	no	no	no	no	yes
K1 XIV	3	M	20-30	no	no	no	no	no
K1 XIV	4	F	40+	no	no	no	no	yes
K1 XIV	8 (Sk.1)	F	30-40	no	no	no	no	yes
K1 XIV	9	F	40+	yes	no	no	yes	yes
K1 XIV	10	F	20-30	no	no	no	no	no
K1 XIV	11	F	40+	no	no	no	no	yes
K1 XIV	18	M	40+	yes	no	no	yes	yes

K1 XIV	26	M	30-40	no	no	no	no	yes

Supplementary Table S4: Dentoalveolar pathologies distribution by sex and age in the Middle Neolithic sample (K1 XII).

	**Females**			**Males**			**TOT**		
**Age class**	**TOT**	**affected**	**% affected**	**MM TOT**	**MM affected**	**% MM affected**	**TOT**	**affected**	**% affected**
20-30	5	0	0	1	0	0	6	0	0
30-40	8	8	100	16	2	12.5	24	10	41.7
40+	18	11	61.1	9	7	77.8	27	18	66.7
**TOT**	31	19	**61.3**	26	9	**34.6**	57	28	**49.1**
